# Bidirectional dispersals during the peopling of the North American Arctic

**DOI:** 10.1038/s41598-023-28384-8

**Published:** 2023-01-23

**Authors:** Javier Rodriguez Luis, Leire Palencia-Madrid, Ralph Garcia-Bertrand, Rene J. Herrera

**Affiliations:** 1 Area de Antropología, Facultad de Biología, Universidad de Santiago de Compostela, Campus Sur s/n, 15782 Santiago de Compostela, Spain; 2 BIOMICs Research Group, Dpto. Z. y Biologia Celular A., Lascaray Research Centre, University of the Basque Country, UPV/EHU, Vitoria-Gasteiz, Spain; 3 Department of Molecular Biology, Colorado College, Colorado Springs, CO 80903 USA

**Keywords:** Biochemistry, Evolution, Genetics

## Abstract

It is thought that Paleo-Inuit were the first people that settled the American Arctic about 5000 BP (before the present) from a migration that crossed Beringia from Northeast Asia. It is theorized that this group initially migrated to the North Slopes of Alaska and subsequently expanded eastward, eventually reaching Greenland. A second circumpolar dispersal of Neo-Inuit from the North Slopes associated with the Thule-Inuk culture has been postulated to have extended eastward around 800 BP, totally replacing the original Paleo-Inuit without admixing. Although generally accepted, this migration scenario is incompatible with previously reported indications of east to west gene flow across the American Arctic. Here we report on the Y-chromosome haplogroup and Y-STR diversity of the four circumpolar populations of the Tuva Republic (N = 24), Northeast Siberia (N = 9), Bethel, Alaska (N = 40), and Barrow, Alaska (N = 31). Four haplogroup lineages (Q-NWT01, Q-M3, Q-M346, and Q-M120) were detected, Q-NWT01 and Q-M3 being the most abundant at 11.11 and 66.67% in Northeast Siberia, 32.50 and 65.00% in Bethel, and 67.74 and 32.26% in Barrow, respectively. The same samples genotyped for Y-chromosome SNPs were typed for 17 Y-STYR loci using the AmpFlSTR Yfiler system. Age estimates and diversity values for the Q-NWT01 and Q-M3 mutations suggest extensive movement of male individuals along the entire longitudinal stretch of the American circumpolar region. Throughout the entire region, Q-M3 exhibits a west to east decreasing gradient in age and diversity while Q-NWT01 indicates the opposite with older TMRCA and higher diversity values running from east to west with the most recent estimates in Canada and Alaska. The high age and diversity values in Greenland are congruent with an origin of the Q-NWT01 mutation in the east of the circumpolar range about 2000–3000 ya. This scenario is incompatible with a complete biological replacement starting about 700 BP of Paleo-Inuit like the Dorset by the Thule-Inuit (Neo-Inuit), as is currently thought, and more parsimonious with gene flow carrying the NWT01 mutation from a pre-Thule population to the ancestors of the present-day Inuit.

## Introduction

### The complex history of the human settlement of America

Anthropological and genetic data indicates that the New World was populated from northeastern Asia in a series of waves^1^. A window of opportunity for terrestrial migration from Northeast Asia towards the New World opened between ~ 30,000 ya (years ago) to ~ 16,000 ya when a drop in global temperatures caused glacial build-up and a drop in sea level. These changes led to creation of a land bridge known as Beringia^2,3^. Yet, glaciers prevented the Beringian migrant population from moving eastward into America or westward into Asia, and the migrants became trapped on the land bridge^4^. At approximately 18,000 ya a period of global warming commenced, and the sea levels began to rise as glaciers melted, allowing the movement of people into North America^5,6^. It is generally thought that this population eventually gave rise to the Amerind natives of north and south America^7,8^. More recently, about 9000 ya, a different group of people who probably had ancestry with groups that lived in Lake Baikal area in Northeast Asia migrated into North America, the ancestors of the Na-Dene people^9^. This dispersal expanded eastward into Alberta, Canada and southward along the British Columbia coast, eventually populating part of Southwest United States, today represented by groups such as the Apache and Navajo^10^. A third major dispersal from the Chukotka area occurred about 5000 before the present (BP)^11^. It is postulated that this group initially migrated to the North Slopes of Alaska ~ 5000 BP from Northeast Asia, developed the Arctic Small Tool tradition and subsequently its descendants expanded eastward^12^. A second circumpolar dispersal associated with the Thule culture dispersed eastward from the North Slopes around 800 BP^11^.

### Cultural sequence

The earliest identified Inuk group, the Early Paleo-Inuit, date back to about 5000 BP in the North Slope of Alaska^11^. Although the tradition evolved in the New World, its origins are thought to reside in Northeast Siberia rooted in Neolithic cultures^13^. Early Paleo-Inuit were the first group of people to live in the North America Arctic exposed to severe cold, very limited seasonal food resources and scarcity of fuel and raw materials^14^. Members of the Norton culture, an Early Paleo-Inuit group, hunted for large sea mammals on the open sea ice^2^. They practiced a flexible economy and were able to colonize rapidly a vast territory from northern Alaska across Arctic Canada into northern Greenland, 5000 km east to west and 3000 km north to south^15^.

Since then, several cultures have developed under two major traditions, the Paleo-Inuit and Neo-Inuit. During the period spanning 5000–2800 BP cultures such as the Denbigh Flint Complex, Pre-Dorset, Early/Middle/Lare Dorset, Independence I, Saqqaq, Choris and Norton under the Early Paleo-Inuit tradition are recognized^16^. The Choris (3600–2500 BP), Norton (3000–1200 BP) and Ipiutak (2400–1200 BP) cultures appear in northern Alaska^2,17^. About the same time in the eastern Arctic region (eastern Canadian Arctic and Greenland), the Dorset tradition emerged and persisted from ~ 2800 to 700 BP^18^. The Dorset used triangular end-blades, soapstone oil lamps and burins. It has been theorized that the Dorset developed from one or several Early Paleo-Inuk traditions, such as the Pre-Dorset, Saqqaq or Independence I, although the earlier cultures had bow and arrow as well as drilling technology, which the Dorset lacked. The Dorset vanished about 700 BP coinciding with the arrival from the Bering Strait area of a culturally different group of people, the Thule^19^.

The Thule culture differs from the Dorset in their dependence on whale hunting for their subsistence^19,20^. It is theorized that the Thule people were the descendents of the Birnirk culture of Chukotka^19^. With the Thule culture came more effective means of transportation, including dog sleds and large skin boats, complex tool kits like sinew-backed bows and harpoons, float gear for hunting large whales and sinew-backed bows^19^. The most recent estimates indicate that the Thule-Inuk culture migration started about 800 BP and spread quickly throughout the eastern Arctic, rapidly replacing the Dorsets in most regions^20^.

### Genetic continuity versus discontinuity?

One of the central issues that have captured the interest of investigators in the study of the settlement of the American Arctic is whether genetic continuity occurred among the various Inuk cultures, specifically the Paleo-Inuk and Neo-Inuk cultures. Controversy still exists regarding whether contemporary Inuk groups entirely derive from Thule populations that migrated across the Arctic about 800 BP^21^ or are the result of admixture events involving Dorset populations and Thule groups^22^. Most of the reports based on uniparental^23^ and genome-wide genetic markers support a total replacement scenario without admixture involving Paleo-Inuit and Neo-Inuit as the latter migrated eastward from North Alaska to Greenland about 800 BP^24,25^. Instances of technology transfer, such as the Thule adoption of Dorset harpoon-head styles, have been attributed to the salvage of remains from abandoned Dorset sites^19^.

Some reports involving mtDNA haplogroups and genome-wide sequences suggest genetic discontinuity between Paleo-Inuk and Thule populations dating to the replacement period (~ 700 BP) but provide evidence of ancient admixture events in Northeast Siberia around 4000 BP between the ancestors of Paleo-Inuit and Neo-Inuit^11^. In one report, mtDNA haplogroup D2a1, abundant in the Saqqaq and Dorset in Paleo-Inuit of Greenland, but totally absent in Neo-Inuit, supports the idea that the ancestors of the two cultures entered the North American Arctic from Northeast Siberia as a single migration around 4000 BP and remained isolated until their replacement by the Thule about 700 BP^19^. Simulation analyses performed in the same study using genomic sequences from a high-coverage of a 4000-year-old Saqqaq individual also demonstrate that admixture likely occurred from the Neo-Inuk lineage into the Saqqaq, likely in Northeast Siberia. More recent studies based on genomic data using rare allele and haplotype sharing indicate that genetically Paleo-Inuit and Thule Neo-Inuit are closely related, but not identical^21^. Two years later Flegontov and collaborators demonstrated that Neo-Inuit from the American Arctic and Northeast Siberia inherited many of their genes from Paleo-Inuit, and the authors suggested that the Neo-Inuk migrants in the American Arctic region are genetically linked to a single Paleo-Inuit Siberian source^12^.

Not all available data suggest a total replacement of Dorset by Thule populations. Several reports provide data in favor of recent gene flow between Paleo-Inuit and Neo-Inuit. For example, studies that explored the mtDNA make-up of contemporary Inuk populations of Canada and Greenland detected diverse complex distribution of A2b founder haplotypes difficult to explain by just an advancing Thule expansion. The observed complex A2b haplotypic distribution is more parsimoniously explained by a matrilineal contribution to contemporary Inuit populations by the Dorset peoples who inhabited Greenland and the Canadian Arctic prior to the Thule expansion^22^. Also, mtDNA data based on A2, A2a, A2b, D4b1a, and D2a haplogroups indicate the presence of basal lineages in the Alaskan North Slope, which is consistent with northern Alaska as the starting point for both Paleo- and Neo-Inuk migrations eastward into Canada and Greenland. The study also detected haplogroup D2a, previously seen only in Aleuts and Paleo-Inuit, in the Alaskan North Slope, suggesting genetic continuity between Paleo-Inuit and contemporary Inuit in this region^26^. Also, Y-chromosome results from contemporary Greenland Inuit under the Q-NWT01 sub-haplogroup suggest that these individuals may be descendents of the Dorset Paleo-Inuk culture and thus indicating the possibility that the contemporary population of Greenland represents admixture between Dorset Paleo-Inuit and Thule Neo-Inuit^27^. In addition, radio-carbon dates from the Canadian Arctic indicate 50–200 years of temporal and regional coexistence involving Dorset and Thule groups that may have provided opportunity for gene flow^20^. It is likely that the genetic data presented above arguing for and against admixture between Thule and Dorset populations represents differences in the marker systems used, regional differences, sex bias gene flow and/or errors introduced by limited number of subjects examined. At this stage, it is difficult to access the true underlining reasons.

### Aims of the study

Two Y-chromosome haplogroups, Q-M3 and Q-NWT01, predominate in the American circumpolar populations that have been studied^28^. However, only populations from the North West Territories of Canada^29^ and Greenland^28^ have been previously phylogenetically examined. Q-M3 decreases in frequency in groups from Greenland compared to the North West Territories of Canada while Q-NWT01 exhibits the opposite distributions^27^. It has been suggested that the Paleo- and Neo-Inuk cultures originated in the Bering Strait region^20^. Thus, despite the fact that knowledge of the Alaskan distributions of the Q-M3 and Q-NWT01 haplogroups is paramount for a complete understanding of the genetic origins of the Yupik and Inuit, this geographic region has not been studied. To address this gap in our understanding of these populations, we analyzed Y-chromosome haplogroup and Y-STR data to address the informational lacuna that exists regarding the peopling of the Alaskan circumpolar region. Together with the previously published Y-chromosome data, these analyses indicate that a west to east decreasing gradient exists in the frequency and diversity of Q-M3 values. We also sought to determine if the decreasing trend in frequency and diversity values of Q-NWT01 in the North West Territories of Canada compared to Greenland that has been previously reported also extends into Alaska.

## Materials and methods

### Samples

**Figure 1:**
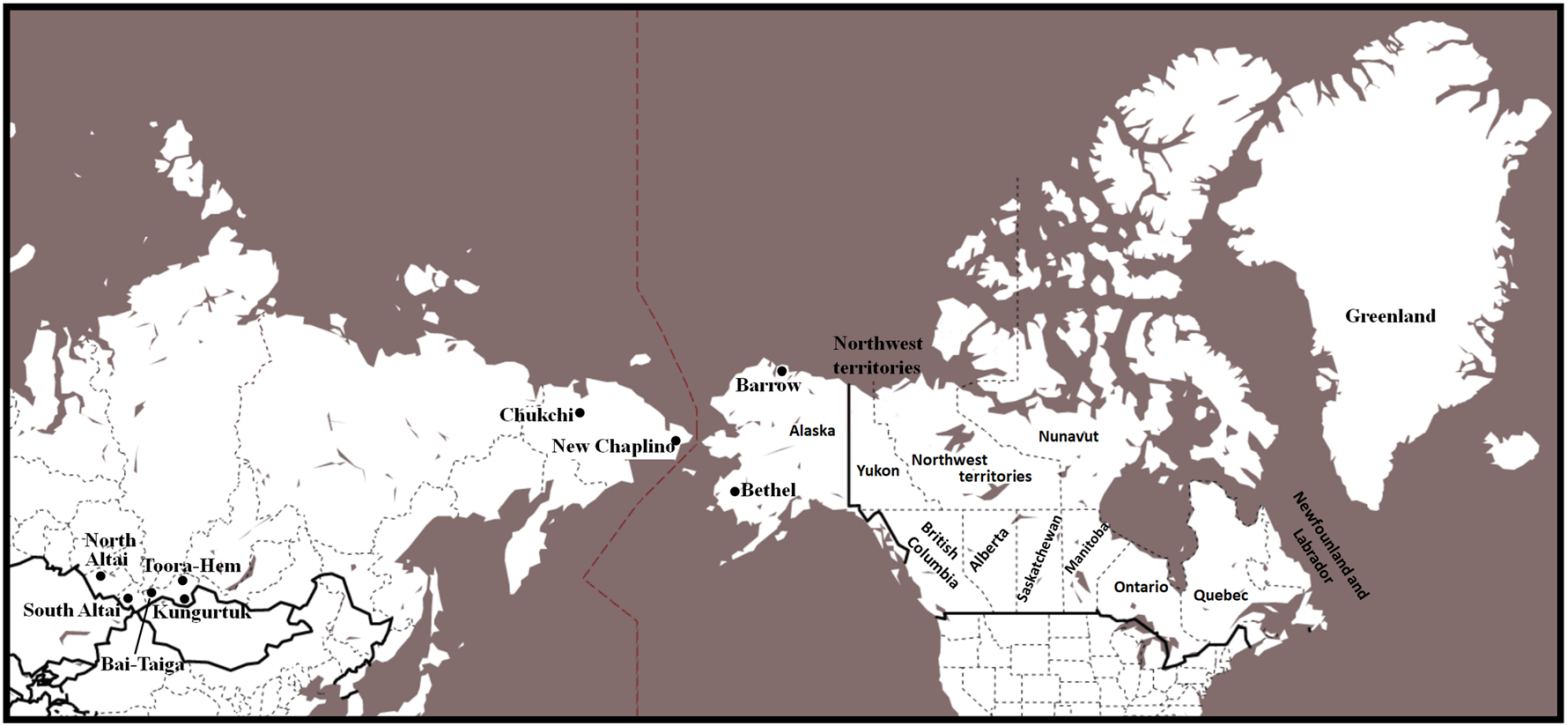
Geographical location of populations examined.

A total of 104 male individuals belonging to seven populations from Central Asia, Northeast Asia and Alaska were found to belong to Y-chromosome haplogroup Q-M242. The populations from Asia include three from the Tuva Republic in Central Asia (Bai-Tai, Kungur-tug, and Toora-Hem, n = 24) and two from the northeast tip of Siberia (New Chaplino and Chukchi, n = 9). The Alaskan populations include one from Bethel in West Alaska (Yupik, n = 40) and one from Barrow in the North Slope (Inuit = 31). The geographic locations of the above-mentioned groups in addition to the reference populations are illustrated in Fig. 1. The populations examined, number of individuals belonging to haplogroup Q and references to previously published populations used in the phylogenetic analyses are presented in Supplementary Table 1. Genealogical history was recorded from each donor for at least two generations to verify lack of familial relationship among donors and their autochthonous origin to the region from both paternal and maternal lineages.

### Compliance with ethical standards

Following the ethical guidelines stipulated by the research institutions involved in this project, all samples were procured voluntarily and with informed consent while closely adhering to the ethical guidelines stipulated by Colorado College, Colorado Springs, Colorado USA. The study adhered to the tenets of the Declaration of Helsinki for the protection of human subjects. The IRB of Colorado College approved this study. All experimental protocols were approved by the IRB of Colorado College.

### DNA extractions and storage

DNA extraction from blood was performed using the standard phenol–chloroform method and ethanol-precipitated as described previously^30^. A NanoDrop 1000 Spectrophotometer (Thermo Scientific) was used for DNA quantitation. Samples were stored as stock solutions in 10 mM Tris–EDTA at − 80 °C.

### Y-chromosome haplotyping

**Figure 2:**
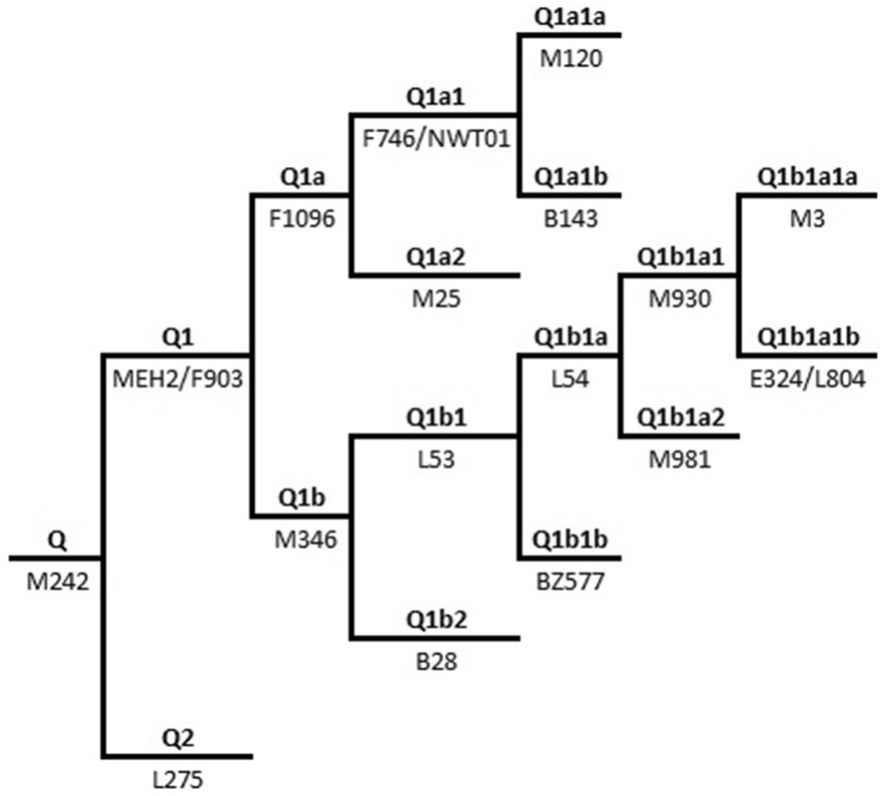
Phylogenetic relationship of Y-SNP markers.

A total of six Y-chromosome bi-allelic markers, Q-M242, Q-MEH2, Q-M120, Q-M346, Q-M3^31^ and Q-NWT01^29^, were hierarchically genotyped by standard methods. These methods included: PCR/RFLP, sequencing and allele specific PCR^32^. The phylogenetic relationship of these markers and the haplogroups they represent are included in Fig. 2. Haplogroup designations are in accordance with the International Society of Genetic Genealogy (2020). The most current version of the Y-DNA Haplogroup Tree was employed and can be found at http://www.isogg.org/tree/ (International Society of Genetic Genealogy. 2020. Y-DNA Haplogroup Tree 2020, version: 15.73. 11 July 2020).

DNA samples with a Q-M242 background were also typed for 17 Y-STR loci (DYS19, DYS385a/b, DYS389I, DYS389II, DYS390, DYS391, DYS392, DYS393, DYS437, DYS438, DYS439, DYS448, DYS456, DYS458, DYS635, GATA H4) using the AmpFlSTR Yfiler kit (Applied Biosystems, Foster City, CA). PCR was performed as per manufacturer’s (Applied Biosystems: AmpFlSTR Yfiler PCR amplification kit user’s manual. 2021. 850 Lincoln Center Drive, Foster City, CA 94404, USA. www.appliedbiosystem.com) specifications. The PCR products generated were separated in an ABI Prism 3130xl Genetic Analyzer and the Amplicon sizes and alleles designations were assess using the GeneMapper v 6.0TM program.

### Data availability

All the data generated in this study, which includes the Y-STR genotypes within the Q haplogroup and the Y Q sub-haplogroups are provided in Supplementary Table 3.

### Data analyses

Y-chromosome STR haplotypes of haplogroup Q males from a total of 8 previously published, geographically targeted populations (Supplementary Table 1) were chosen for phylogenetic comparison. A Multidimensional Scaling (MDS) analysis was performed at the level of populations (SPSS v.20) using Rst distances estimated from haplogroup Q Y-STR haplotypes^33^. The pairwise population comparisons were tested at a significance level of 0.05 with 10,000 permutations. To compensate for potential inclusion of false positives, type I statistical errors, the Bonferroni correction was applied (α/m = 0.05/66 = 0.00076). DYS385 was excluded from the haplotype diversity calculations because it is not possible to discriminate between the DYS385a and DYS385b loci with the Y STR kit. The number of repeats at DYS389II was calculated by subtracting the number of repeats at DYS389I. Discrimination capacity was calculated by dividing the number of different haplotypes by the total number of individuals in the population. The fraction of unique haplotypes was determined as the percent proportion of unique haplotypes.

Median-joining (MJ) networks^34^ based on Y-STR profiles of individuals possessing the Q-M242, Q-M346, Q-M3 and Q-NWT01 mutations were constructed with the NETWORK 4.5.1.6 software (www.fluxus-engineering.com), in which the Y-STR markers were weighted inversely to their repeat variance. Average gene diversity (GD) based on Y-STR loci were computed according to Nei^35^. Intra-haplogroup diversity (mean microsatellite variance: Vp)^36^ was calculated across 15 loci for each Q haplogroup and relevant population. Y-STR haplotypes were used to estimate the time to the most recent common ancestor (TMRCA). With this aim, rho statistic (*ρ*)^22^ and weighted rho (*ρ*_W_)^37^ were estimated with an R script available in GitHub (http://github.com/fcalafell/weighted_rho). Mutation rates were obtained from the Y-Chromosome STR Haplotype Database (YHRD, www.yhrd.org) in March 2020. The statistical significance of the time estimate differences was assessed using the Past 4.02 software (http://palaeo-electronica.org/2001_1/past/issue1_01.htm).

## Results

### Distribution of Y-chromosome Q sub-haplogroups

Considering the significance of haplogroup Q in the study of Paleo-Inuk and Neo-Inuk dispersals into the New World, we typed members of this lineage by high-resolution SNP analysis using six bi-allelic markers to better understand the genetic relationships between Old and New World populations and the migrations that connect them. Figure 2 illustrates the phylogenetic relationships of the six Y-SNPs genotyped within haplogroup Q while Supplementary Table 2 provides the frequencies of the sub-haplogroups observed in the four Asian and American populations from the Tuva Republic, Northeast Siberia, Barrow and Bethel genotyped in the present study. A total of 104 Q Y-chromosomes were examined and although specific markers predominate in each of the groups examined (*ie*, Q-M346 in Tuva, Q-M3 in Northeast Siberia, Q-NWT01 in Barrow and Q-M3 in Bethel), the four populations are not monomorphic in their sub-haplogroup constitution (Supplementary Table 2).

All Q Y-chromosomes in this study were derived for the Q1-MEH2 lineage (Fig. 2). In the three populations examined from the Tuva Republic, all individuals were found to be derived for Q-M346 except for one sample from Kungur-tug, which was Q-M120. In far Northeast Siberia in the Chukchi Peninsula, the Q-M3 lineage predominates with only two Q-M346 and one Q-NWT01 individuals. On the American side of the Bering Strait, in the Yupik of Bethel, Western Alaska, sub-haplogroup Q-M3 remains dominant at a frequency of 65.00% (n = 26) while Q-NWT01 is derived in the rest of the population except one sample derived for Q-M346. In the North Slope of Alaska, in the Barrow Inuk population, the opposite distribution relative to Bethel is observed with 67.74% possessing the Q-NWT01 lineage with the rest of the individuals being Q-M3. No other lineage was detected in the Barrow group.

### Haplotype diversity

The analyses of the 17 Y-STRs provided additional details on the paternal diversity within the Q haplogroup. Haplotypes for the 104 haplogroup Q individuals are available in Supplementary Table 3. Overall, we identified 45 distinct haplotypes at the 17- loci Y STR resolution, 10 from the Tuva Republic, 5 from Northeast Siberia, 17 from Bethel and 13 from Barrow (Supplementary Table 3). The microsatellite diversity for each population reflects the heterogeneity observed in the number of haplotypes (Supplementary Table 3). The highest microsatellite diversity within haplogroup Q at the 15-loci Y-STR resolution is observed in Northeast Siberia (New Chaplino/Chukchi) (Vp = 0.6778). Several haplotypes are recurrent, especially in the populations of Barrow and Bethel (Supplementary Tables 3). No haplotypes are shared among populations except for 5 haplotypes between the Bethel and Barrow populations (Supplementary Table 3). The Y-STR allelic frequencies for the populations of Barrow, Bethel, Northeast Siberia, and the Tuva Republic are provided in Supplementary Tables 4, 5, 6 and 7, respectively.

### Population relationships based on Haplogroup Q

**Figure 3:**
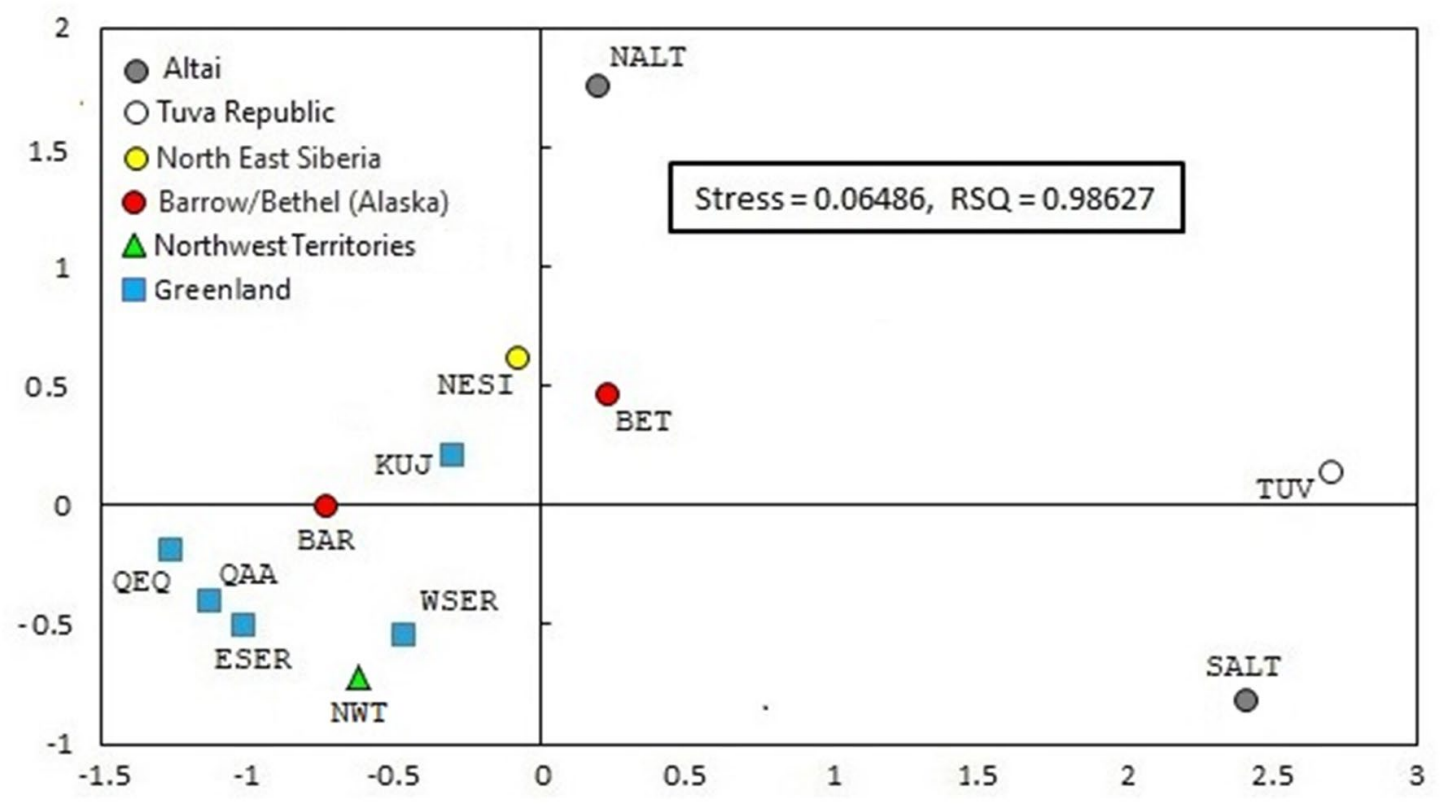
MDS plot of populations examined.

Rst pairwise genetic distances among genotyped and reference populations were calculated based on 15-loci Y-STRs and are provided in Supplementary Table 5. The distances and segregation among the populations are visualized in a MDS plot (Fig. 3). In this two-dimensional graph (stress value = 0.06486, R^2^ value = 0.98627), the groups partition diagonally with the two dimensions contributing to the separation of the populations. Quadrant III of the graph exhibits a cluster made up of all but one (Kujalleq) of the Greenland Inuit communities, which plots near in quadrant II. The Barrow Inuit of the Bering Strait region and the North West Territories Inuit from Canada partition in close proximity to this Greenland cluster. The Northeast Siberia groups of Chukchi/New Chaplino and the Yupik of Bethel partition close by in the diagonal line. More distant diagonally in quadrant I, the North Altai samples separate. The Tuva and South Altai populations separate distantly from the rest of the populations along the first dimension in quadrant I and IV, respectively. It is evident from the plot that an affinity exists among the populations from Greenland, North West Territories and Barrow, which is considerably less relative to Northeast Siberia and Bethel, which plot close to each other.

### Network analysis

**Figure 4:**
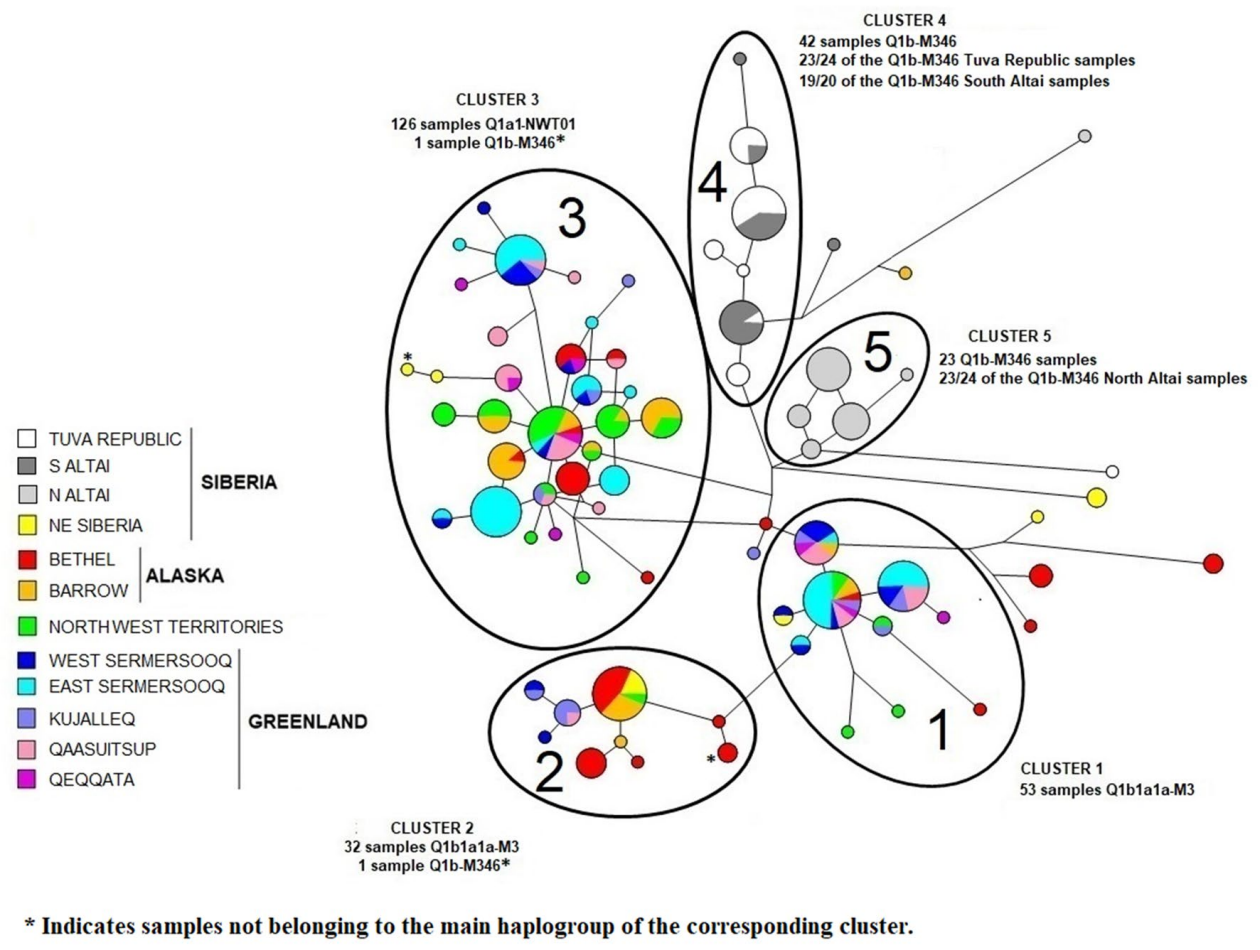
Network analysis based on haplogroup Q Y-STR loci.

The network analysis based on 15-loci Y STR haplotypes within Haplogroup Q including sub-haplogroups Q1b-M346, Q1b1a1a-M3 and Q1a1-NWT01 is illustrated in Fig. 4. Five clearly delineated clusters are evident. Clusters 1 and 2 are almost entirely made up of Q-M3 individuals. The Q-M3 samples from East Sermersooq, Greenland and North West Territories, Canada are found in cluster 1, while the Q-M3 samples from Bethel, Alaska partition mainly into cluster 2. Cluster 3 is overwhelming Q-NWT01 from all the circumpolar American populations and Northeast Siberia. Clusters 1, 2 and 3 exhibit star-like topologies. Cluster 4 contains most of the samples from the Tuva Republic and South Altai, all Q-M346, while in cluster 5 most of the individuals are Q-M346, all from North Altai. It is noteworthy that cluster 4 exhibits considerable inter-population sharing involving South Altai and Tuva Republic individuals suggesting genetic affinity between the two populations, while cluster 5 is restricted to North Altai samples indicating Y-chromosome dissimilarity to the South Altai and Tuva Republic populations. No population sub-structure is evident within each of the clusters. Intra- and Inter-population haplotype sharing is prevalent except within cluster 5. At times inter-population haplotype sharing included populations expanding the entire circumpolar region examined (Fig. 4).

To examine in more detail, the relationships among individuals within the two predominant American circumpolar sub-haplogroups, Q-M3- and Q-NWT01-specific network analyses were performed. Supplementary Fig. 1 illustrates the network analysis of the Q-M3 samples. Sub-population structure is observed in the network involving individuals from Greenland, which segregate into groups with identical and similar haplotypes. Similarly, the samples from Bethel tend to partition in proximity to each other. Intra- and inter-population haplotype sharing is also observed especially involving samples from Greenland. The larger number of Greenland individuals examined may be a contributing factor. The Q-M3 samples partition bipolarity with about one half of the samples exhibiting a star-like topology with a central node and satellite haplotypes mostly made up of individuals from Greenland. This central node also includes samples extending the entire American circumpolar region from Greenland to Barrow and Bethel of Alaska. Most of the individuals from Bethel and Barrow in Alaska segregate into the second half of the network. Several samples from Greenland are also located within this second cluster. The samples from Northeast Siberia are in both halves of the network. The Northeast Siberia individuals that partition with most of the samples from Greenland segregate distant from the central node, separated by many mutational steps (2–13). The Northeast Siberia samples located in the other half of the network have sequence similarities with the haplotypes from Barrow and Bethel.

The Q-NWT01 network exhibits a star-like topology with a central node made up of all the American circumpolar populations (Supplementary Fig. 2). From this major central node, several smaller satellite nodes branch-out, one mutational step away, exhibiting intra- and inter-population haplotype sharing involving all possible combinations of populations. Overall, the number of instances of haplotype sharing exceeds the number of singletons, an indication of limited diversity at the Y-STR level. Most of the haplotypes within the network are separated from each other by only one mutational step. Except for some individual from Greenland, which partition distantly in a separate cluster, no sub-population structure is observed in the network. The samples from Greenland exhibit a greater degree of intra-population haplotype sharing compared to the other groups.

### Time estimates and diversity

Table 1 includes the coalescence time estimations based on 15 Y-STR loci for sub-haplogroups Q1b-M346, Q1b1a1a-M3 and Q1a1-NWT01 for the populations examined in this study. The time estimations of the populations from the Tuva Republic, Northeast Siberia, Barrow and Bethel of Alaska were obtained from Y-STR and Y-SNP genotypes generated in this study while the corresponding data for the populations of the North West Territories and Greenland were calculated from data from the original studies^27,29^, respectively. Due to the limitations and assumptions associated with the current calibrations of Y-STR mutation rates^38–41^, as well as differences in methodology among studies in estimating diversity and ages, the dates generated in this study should only be taken as relative estimates. Nevertheless, these relative values may be useful for comparisons among the circumpolar populations studied in this report.

**Table 1:** Age estimates.

		Rho (R script "weighted age" F. Calafell)				ASD (Kilin-Klyosov TMRCA calculator)	
		Age		Weighted age			
		Years	SD	Years	SD	Years	SD
**Tuva Republic**	25 years/generation	951	238	783	225	899	263
[Q-M346]	30 years/generation	1141	285	940	271		
n = 23 (95.83% of the population)		rho: 1.3913		rho: 1.1453			
**North East Siberia**	25 years/generation	3760	702	7114	1037	8407	1778
[Q-M3]	30 years/generation	4512	843	8537	1245		
n = 6 (66.67% of the population)		rho: 5.5		rho: 10.4067			
**Barrow**	25 years/generation	1139	389	1248	379	2381	689
[Q-NWT01]	30 years/generation	1367	467	1497	455		
n = 21 (67.74% of the population)		rho: 1.6667		rho: 1.8253			
**Barrow**	25 years/generation	2507	603	3940	725	3947	1582
[Q-M3]	30 years/generation	3008	723	4727	870		
n = 10 (32.26% of the population)		rho: 3.6667		rho: 5.7629			
**Bethel**	25 years/generation	1104	328	1456	355	1473	686
[Q-NWT01]	30 years/generation	1325	394	1747	426		
n = 13 (32.50% of the population)		rho: 1.6154		rho: 2.1296			
**Bethel**	25 years/generation	2677	382	4998	521	5999	1526
[Q-M3]	30 years/generation	3213	459	5997	625		
n = 26 (65.00% of the population)		rho: 3.9167		rho: 7.3106			
**Greenland**	25 years/generation	1960	410	2072	385	3391	940
[Q-NWT01]	30 years/generation	2352	492	2487	462		
n = 68 (57.14% of the population)		rho: 2.8676		rho: 3.0315			
**Greenland**	25 years/generation	764	171	1367	223	2067	727
[Q-M3]	30 years/generation	917	205	1640	268		
n = 51 (42.86% of the population)		rho: 1.1176		rho: 1.999			
**North Western Territories**	25 years/generation	1052	252	1262	265	1887	622
[Q-NWT01]	30 years/generation	1262	303	1514	318		
n = 26 (81.25% of the population)		rho: 1.5385		rho: 1.8461			
**North Western Territories**	25 years/generation	1595	426	2343	517	3544	938
[Q-M3]	30 years/generation	1914	512	2812	620		
n = 6 (18.75% of the population)		rho: 2.3333		rho: 3.4277			

Q-M346 is the most abundant lineage (95.83%, 23/24 individuals) in the Tuva Republic (Supplementary Table 2). Although the origin of Q-M346 dates back to 24,000–27,900 ya in Eurasia^42^, we found that in the Tuva Republic the Q-M346 lineage only dates to 783–940 years (25- and 30-years generation time, respectively) using the weighted age method of Calafell and 899 years with the Kilin-Klyosov method (Table 1). The micro-satellite diversity within haplogroup Q is commensurately low (Vp = 0.1705). In fact, when compared to the other genotyped population, the Tuva Republic exhibits the lowest diversity value within haplogroup Q compared to the rest; Northeast Siberia the highest (Supplementary Table 8). Based on these values, it is likely that the population of the Tuva Republic represents a relative recent replacement of an earlier/older population by Q-M346 individuals.

Based on six samples, the age estimate of Q-M3 in Northeast Siberia (7114–8537 ya) is considerably older than the Inuit of Barrow (3940–4727 ya) and the Yupik of Bethel (4998–5999 ya) in Alaska (Table 1). Congruently, the microsatellite diversity (Vp) for Q-M3 is higher in Northeast Siberia (Vp = 0.4689) than in Barrow (0.2093) and Bethel (0.2884) (Supplementary Table 10). Except for gene diversity in Bethel (0.9508), diversity is greater in Northeast Siberia than in Alaska (Barrow and Bethel). The age estimates of Q-M3 in the Inuit of the North West Territories of Canada (2343–3544 ya) is lower compared to Barrow and Bethel while its Vp (0.2089) value is lower than in Bethel, but comparable to Barrow. In Greenland, overall, the age estimates values (1367–2067 ya) and diversity (Vp = 0.1024) drop even lower compared to the North West Territories. The standard deviations do not overlap among the age estimate values of the populations mentioned above, except for the older age values of Barrow and North West Territories. Conversely, when the Q-NWT01 lineage is examined, the oldest age estimates (2072–3391 ya) and higher diversity value (Vp = 0.2805) are found in Greenland at the eastern end of the longitudinal circumpolar axis. The age estimates in the North West Territories are 1262–1887 ya and the Vp = 0.1422. Although the population of Bethel exhibits comparable age estimates in relation to North West Territories, its diversity value (Vp = 0.1051) is less than in North West Territories. The population of Barrow exhibits comparable age and diversity values relative to North Western Territories (Table 1 and Supplementary Table 10, respectively). When the two indexes of diversity (gene diversity and Vp) for both Q-M3 and Q-NWT01 lineages are compared among the various communities within Greenland, the values fluctuate independent of geographic location within the island, likely resulting from the small population size sampled and/or random drift.

## Discussion

Although previous investigations have been conducted on communities from the eastern portion of circumpolar America including Greenland^28^ and Northwestern Canada^29^, no comparable Y-chromosome phylogenetic analyses exist on populations from the western regions of the Americas, specifically Alaska. The Y-chromosome Y-SNP and Y-STR profiles of the circumpolar populations examined in the present study illustrate a region recently populated (~ 6000 BP) by migrants from Northeast Asia. The Y-chromosome haplogroups and haplotypes observed throughout the American circumpolar region from Alaska to Greenland exhibit strong genetic affinities with the ones in Northeast Siberian populations just across the Bering Strait in the Chukchi Peninsula. Yet, the Y-chromosome similarities with groups further west in the Altai and Tuva regions are limited.

The Y-chromosome differences between the populations in the Altai/Tuva region and the Inuk populations of the Americas is reflected at the haplogroup level in the almost complete presence of the Q-M346 haplogroup in the former and the dominance of Q-M3 and Q-NWT01 in the latter. In the extreme Northeast Asia in the Chukchi Peninsula, although Q-M3 predominates, Q-M346 and Q-NWT01 were detected as well. Across the American circumpolar region Q-M3 and Q-NWT01 dominate or represent the entire Native Y-chromosome component of the expanse. In coastal west Alaska, the Yupik population of Bethel is 65.0% Q-M3 and 32.5% Q-NWT01 while the Inuit of Barrow in the Northern Slopes are 67.7% QNMT01 and 32.3% Q-M3. In the North West Territories of Canada 81.0% of the Inuit are Q-NWT01 and 19.0% are Q-M3^29^ while in Greenland Q-NWT01 is 57.0% and Q-M3 is 43.0% of the Inuk population^27^.

The Y-STR profiles within the Q haplogroup indicate marked partitioning of haplogroup-specific Y-STRs into separate clusters (Fig. 4). Cluster 1, for example, is entirely made up of Q-M3 individuals, mainly from Greenland. Significantly, samples from the North West Territories of Canada and even from Northeast Siberia are found sharing haplotypes with Greenlanders. In cluster 2, we find an abundance of Q-M3 samples from Bethel, Alaska, and several in a central node exhibiting haplotype sharing with Barrow, North West Territories and Northeast Siberia. The observed split of Q-M3 males into two clusters (1 and 2) is also observed in the Q-M3-specific network (Supplementary Fig. 1) where a bi-polar topology separates most of the Greenland samples from the Bethel/Barrow individuals by a 3-mutation gap. Cluster 3 contains all the NWT01 samples genotyped, with haplotype sharing involving populations encompassing the entire longitudinal stretch of the American circumpolar region. Clusters 4 and 5 have most of the Q-M346 samples. Cluster 4 includes most of the samples from the Tuva Republic and the South Altai, the majority involved in inter-population haplotype sharing. The extensive degree of haplotype sharing throughout clusters 1–3 of haplogroup Q (Fig. 4) and in the Q-M3-specific network (Supplementary Fig. 1) strongly suggest extensive population movement along the circumpolar region from Northeast Siberia to Greenland involving Q-M3 and Q-NWT01 males. Furthermore, the extensive haplotype sharing in large central nodes in stem locations of clusters 1–3 suggest rapid, long-distance dispersal of Q-M3 and Q-NWT01 individuals. This longitudinal movement of people in the northern-most region of America is also reflected in the Q-NWT01-specific network (Supplementary Fig. 2), which shows considerable haplotype sharing (including in the central node in a stem position) involving populations from throughout the circumpolar region, especially Barrow of the Alaskan North Slopes and North West Territories of Canada. This Y-chromosome continuum does not extend to the Altaic region of Asia where the South Altai shares strong affinities with the Tuva Republic and both exhibit disparity from the North Altai.

The Y-chromosome landscape provided by the network analyses is mirrored by the MDS plot based on haplogroup Q Y-STR data (Fig. 3). The MDS graph illustrates the same continuum of circumpolar populations with the Greenland and North West Territories communities at one end of a diagonal partition in which Barrow is embedded within them and Northeast Siberia and Bethel plot at the other end. The MDS plot also indicates the affinity between the Tuva Republic and the South Altai in sharp contrast to the North Altai, which segregate at a distance. And yet, as a group, North Altai in the network (Fig. 4, cluster 5) and in the MDS analysis (Fig. 3) exhibits greater proximity to the Inuk populations.

**Figure 5:**
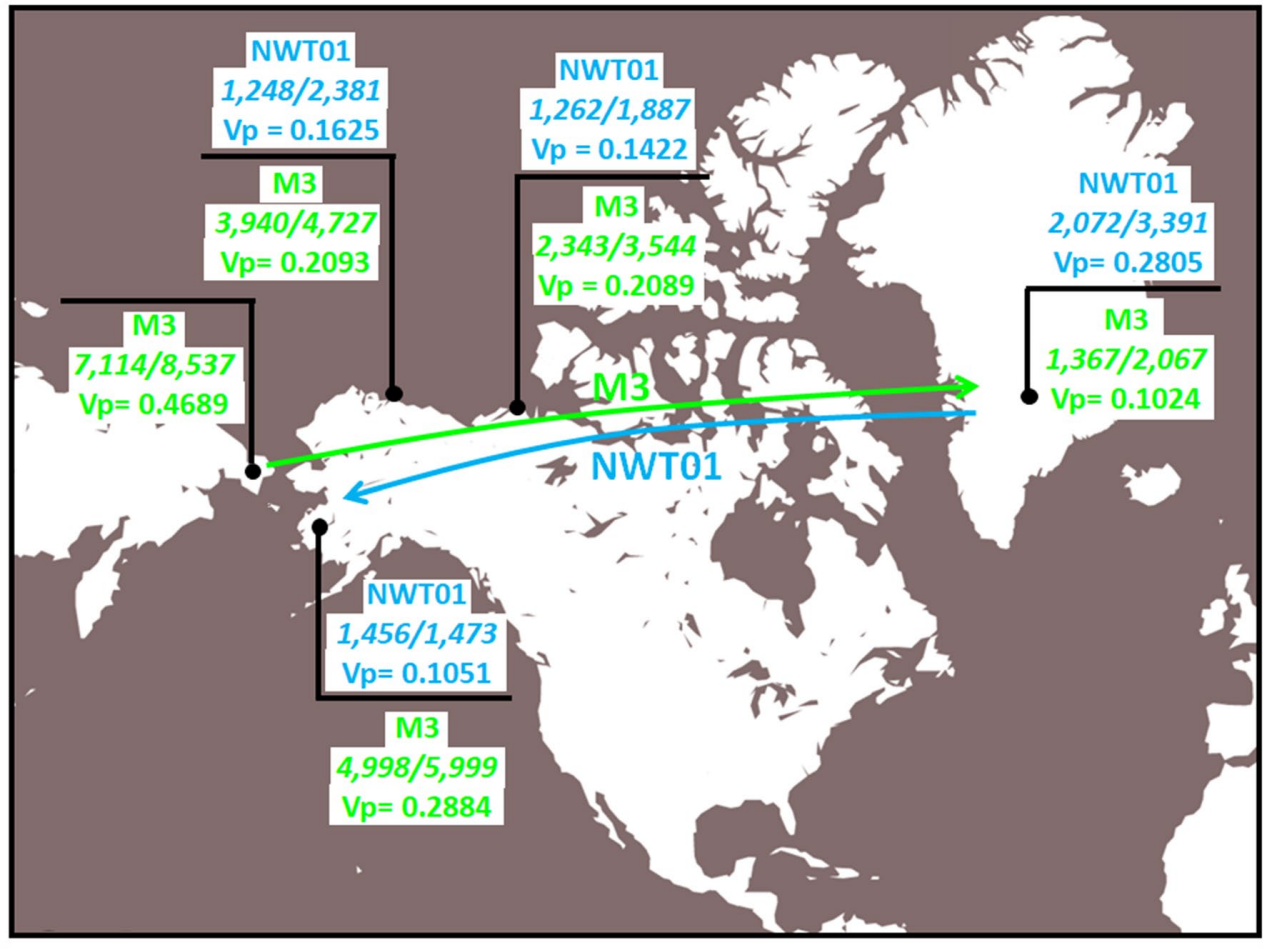
Circumpolar movement of Q-M3 and Q-NWT01 lineages (numbers in italics = age in years).

The age estimates and diversity values provide additional insight into the history of the circumpolar populations. Due to the dominance of the Native Q-M3 and Q-NWT01 lineages in the circumpolar populations, we base our analyses and discussion on these two sub-haplogroups. The communities of Northeast Siberia exhibit the oldest age estimates and highest diversity values of the Q-M3 lineage in the populations examined. Compared to the geographically closest groups of Alaska, some of the age estimates from Northeast Siberia are about twice as old and some of the diversities (Vp) are twice as high (Table 1 and Supplementary Table 10). Although the Northeast Siberia Q-M3 values are based on only 6 samples, these estimates are congruent with a dispersal of Q-M3 individuals across Beringia in a west to east direction, in other words, from Asia to America. Eastward in the communities of the North West Territories of Canada and Greenland, age estimates (Table 1) and diversity values (Supplementary Table 10) continue to drop, with Greenland exhibiting less than 25% the age and diversity compared to Northeast Siberia. This west to east diminution gradient in age and diversity corroborates a human dispersal that may have started in extreme Northeast Asia about 6000 BP, reaching Greenland approximately 2000 BP. In the framework of the archeological record and the theorized recent (starting about 700 BP) biological replacement of the Dorset (Paleo-Inuit) by the Thule (Neo-Inuit), the present study indicating an age gradient that starts about 8000 BP in Northeast Siberia may be explained in terms of a surviving Q-M3 lineage with origins in Asia that is present in the present-day Inuit of the entire American circumpolar region (Fig. 5).

The Q-NWT01 lineage circumpolar landscape is the opposite of the Q-M3 lineage (Fig. 5). The highest age estimates and diversity values are found in the extreme east of the Inuit domain (Table 1 and Supplementary Table 10). The Inuk communities of the North West Territories in Canada register age estimates about 40% younger than Greenland and diversity values that are 50% the values of Greenland. Further west in Alaska, although the populations of Bethel and Barrow generated comparable age estimates and Vp values commensurate with the North West Territories, the gene diversity values are 10–15% less in Alaska. The east to west decreasing trend observed in the estimated ages and diversity of the NWT01 lineage is congruent with a dispersal that was initiated in the east of the circumpolar range about 2000–3000 ya and rapidly extended westward.

It is noteworthy that the age estimates generated by Olofsson and colleages (7000/14,300)^27^ are considerable different from the ones reported in the current study (2072/3391 ya). Even within the same study of Olofsson et al.^27^, an age difference of over 7000 ya was estimated by two different methods. This phenomenon has been previously described and attributed to variation in the calibrations of Y-STR mutation rates as well as differences in methodology among studies^38–41^. Therefore, the age and diversity values reported in this study may be useful only for comparisons among the circumpolar populations studied in this report.

Congruently, the topology of the Q network with all the Q-NWT01 samples grouped into a single compact cluster and the extensive inter-population haplotype sharing, many in stem locations, also suggest that all the Q-NWT01 haplotypes had a common origin and rapidly dispersed among populations connected by gene flow (Fig. 4). This proposed scenario for the origin and dispersal of the NWT01 lineage puts in question the theorized complete biological extinction of the of the Dorset people (Paleo-Inuit) and the total occupation of the Dorset territories by the Thule (Neo-Inuit). Biologically speaking, it is complicated to explain the survival of the NWT01 lineage to the present, which originated prior to the proposed replacement of the Dorset by the Thule. The expectation of such a population replacement starting about 700 BP is that an older Paleo-Inuk (e.g., Dorset) NWT01 lineage would have been eliminated. A likely explanation for this conundrum is the possibility that a Dorset or other Paleo-Inuk population carrying the NWT01 marker interbred with the incoming Thule, thereby passing the mutation to this group. This is not a novel hypothesis since the idea was introduced by Olofsson and colleagues in 2015^27^ in their study of Greenland Inuk communities. The map in Fig. 5 illustrates the hypothetical dispersals of the Q-M3 and NWT01 lineages in opposite directions that we propose, and the corresponding estimated ages and diversities based on the current populations examined. In the dispersal model that we propose both Q-M3 and Q-NWT01 are of Paleo-Inuit origin. An alternative explanation would involve the Q-NWT01 and Q-M3 dispersal eastward as part of the same Thule expansion. It is likely that bottleneck and founding effects could have generated the west to east age and diversity decreasing gradient seen for Q-M3. And, Q-NWT01 chromosomes may have been subject to stochastic fluctuations in diversity generated by dropout events leading to the older ages and higher diversity in the east today. In fact, the work of Raghavan et al.^11^ adds support to this alternative explanation of stochastic fluctuations. The QNWT01 mutation could have originated in the population ancestral to both the Paleo-Inuit and the Thule groups. More recent, or contemporary admixture events that could have transferred the Q-NWT01 mutation from some other present-day group that also carried the mutation to comtempory Inuit are incongruent with the age/diversity estimations and the observed graient. Analyses of archaeological samples of Thule/Punuk/Birnirk individuals would confirm that the Q-NWT01 mutation is not the result of genetic continuity within the Bering Strait and is due to admixture with later Dorset groups. It should be emphasized that the proposed model does not contradict the possibility of ancient admiture events involving the ancestors of Paleo-Inuit and Neo-Inuit in North East Asia as suggested by genome-wide studies^11,19^. Uniparental mutations such as Q-M3 and Q-NWT01 could have been transfer from Paleo-Inuit to Neo-Inuit subsequently and independently. Yet, despite the large contribution to the male line of descent from Paleo-Inuit to Neo-Inuit, so far genome-wide studies show no traces of admixture between the two in the North American Artic. Thus, confirmation of admixture from genome-wide studies should be explore further.

## Conclusion

The Y-chromosome analyses performed in this investigation support the notion that the American circumpolar region was settled by Asian migrants that crossed Beringia and rapidly advanced eastward, reaching Greenland at least 4000 BP. The Q-M3 and NWT01 Y-chromosome lineages predominate in an extensive territory that extend from Northeast Siberia to Greenland. Inter-population haplotype sharing suggests extensive movement of people along the entire longitudinal stretch of the circumpolar region. Throughout the entire American circumpolar range, the Q-M3 lineage exhibits a west to east gradient in age and diversity while the Q-NWT01 lineage indicates the opposite with older TMRCA and diversity values running from east to west with the most recent estimates in Alaska and Canada. High age estimates and diversity values in Greenland suggest an origin of the Q-NWT01 mutation in the eastern portion of the circumpolar range about 2000–3000 ya, a scenario incongruent with a complete biological replacement starting about 800 BP of Paleo-Inuit like Dorset by the Thule (Neo-Inuit) and more parsimonious with gene flow carrying the Q-NWT01 mutation from a pre-Thule population to the ancestors of present-day Inuit. Also, more ancient admixture events in the Western American Artic and subsequent migration carrying the Q-NWT01 mutation eastward are difficult to reconcile with the age/diversity estimations and the observed gradient. Altogether, the evidence presented in this article points to bidirectional gene flow and interbreeding involving Paleo-Inuk and Neo-Inuk populations shaping the present circumpolar populations.

## Supplementary Information

Supplementary Information 1.

Supplementary Information 2.

Supplementary Information 3.

Supplementary Information 4.

Supplementary Information 5.

Supplementary Information 6.

Supplementary Information 7.

Supplementary Information 8.

Supplementary Information 9.

Supplementary Information 10.

Supplementary Information 11.

Supplementary Information 12.

Supplementary Information 13.
