# Supplementary material for: Bidirectional dispersals during the peopling of the North American Arctic
Source: Sci Rep. 2023 Jan 23;13:1268. doi: 10.1038/s41598-023-28384-8 (PMC9871004; doi:10.1038/s41598-023-28384-8)
Supplement: Supplementary file 1 — Supplementary Information 1. [file 41598_2023_28384_MOESM1_ESM.pdf]

Supplementary Table 1. Populations examined.

Population	Code	n	Location	Language	Reference
Siberia					
Tuva Republic	TUV	24	Southern Siberia	Turkic	Present Study
North Altai	NALT	26	Southern Siberia	Turkic	Dulik et al. 2012a
South Altai	SALT	21	Southern Siberia	Turkic	Dulik et al. 2012a
North East Siberia	NESI	9	Northern Siberia	Eskimo–Aleut (Central [illegible]	Present Study
Alaska					
Bethel	BET	38	East Coast, Alaska	Eskimo–Aleut	Present Study
Barrow	BAR	31	North Coast Alaska	Eskimo–Aleut	Present Study
North Western Territories					
North West Territories, Canada	NWT	30	Central American Arctic	Eskimo–Aleut	Dulik et al. 2012b
Greenland					
E Sermersooq	ESER	54	East Coast, Greenland	Eskimo–Aleut	Olofsson et al. 2015
W Sermersooq	WSER	19	West Coast, Greenland	Eskimo–Aleut	Olofsson et al. 2015
Qaasuitsup	QAA	23	West Coast, Greenland	Eskimo–Aleut	Olofsson et al. 2015
Qeqqata	QEQ	8	West Coast, Greenland	Eskimo–Aleut	Olofsson et al. 2015
Kujalleq	KUJ	14	South Coast, Greenland	Eskimo–Aleut	Olofsson et al. 2015

References for populations cited in Supplementary Table 1

1. Dulik MC, Zhadanov SI, Osipova LP, Askapuli A, Gau L, Gokcumen O, Rubinstein S, Schurr TG. Mitochondrial DNA and Y chromosome variation pr[illegible]
2. Dulik MC, Owings AC, Gaieski JB, Vilar MG, Andre A, Lennie C, Mackenzie MA, Kritsch I, Snowshoe S, Wright R, Martin J, Gibson N, Andrews TD, S[illegible]
3. Olofsson JK, Pereira V, Børsting C, Morling N. Peopling of the North Circumpolar Region--insights from Y chromosome STR and SNP typing of Gre[illegible]
