# Supplementary material for: Bidirectional dispersals during the peopling of the North American Arctic
Source: Sci Rep. 2023 Jan 23;13:1268. doi: 10.1038/s41598-023-28384-8 (PMC9871004; doi:10.1038/s41598-023-28384-8)
Supplement: Supplementary file 4 — Supplementary Information 4. [file 41598_2023_28384_MOESM4_ESM.pdf]

Supplementary Table 4. Y- STR allele frequencies in the population of Barrow (n = 30).

Allele	DYS19	DYS389I	DYS389II	DYS390	DYS391	DYS392	DYS393	DYS437	DYS438
10					1.000				0.200
11									0.800
12									
13	1.000	0.100					0.133		
14		0.867				0.333	0.733	0.033	
15		0.033				0.667	0.133	0.967	
16									
17									
18									
19									
20									
21									
22									
23				0.067					
24				0.700					
25				0.233					
29			0.067						
30			0.600						
31			0.333						

DYS439	DYS448	DYS456	DYS458	DYS635	GATA_H4	Genotype	DYS385a/b
					0.700	12.19	0.067
0.467					0.167	13.13	0.033
0.167					0.133	13.18	0.033
0.367						13.19	0.200
			0.667			13,20	0.067
		0.800				13.21	0.267
		0.100	0.133			14.17	0.100
		0.100	0.200			14.18	0.033
	0.667					15.17	0.200
	0.033						
	0.100						
	0.200						
				0.867			
				0.100			
				0.033			
