# Supplementary material for: Bidirectional dispersals during the peopling of the North American Arctic
Source: Sci Rep. 2023 Jan 23;13:1268. doi: 10.1038/s41598-023-28384-8 (PMC9871004; doi:10.1038/s41598-023-28384-8)
Supplement: Supplementary file 5 — Supplementary Information 5. [file 41598_2023_28384_MOESM5_ESM.pdf]

Supplementary Table 5. Y- STR allele frequencies in the population of Bethel (n = 38).

Allele	DYS19	DYS389I	DYS389II	DYS390	DYS391	DYS392	DYS393	DYS437	DYS438
**10**					1.000				0.421
**11**									0.579
**12**									
**13**	0.947	0.316				0.026	0.237		
**14**	0.053	0.658				0.579	0.658	0.158	
**15**		0.026				0.395	0.105	0.816	
**16**								0.026	
**17**									
**18**									
**19**									
**20**									
**21**									
**22**				0.026					
**23**				0.053					
**24**				0.789					
**25**				0.132					
**29**			0.053						
**30**			0.658						
**31**			0.263						
**32**			0.026						

DYS439	DYS448	DYS456	DYS458	DYS635	GATA_H4	Genotype	DYS385a/b
					0.342	11,13	0.026
0.342					0.605	13,13	0.079
0.211					0.053	13,20	0.026
0.263			0.105			13,21	0.158
0.184			0.237			14,17	0.026
		0.289				14,18	0.026
		0.605	0.368			14,21	0.053
		0.105	0.289			15,17	0.053
	0.342					15,18	0.079
	0.026					16,16	0.053
	0.263					16,17	0.184
	0.368					16,18	0.132
				0.895		17,18	0.079
				0.079		17,19	0.026
				0.026			
