# Supplementary material for: Bidirectional dispersals during the peopling of the North American Arctic
Source: Sci Rep. 2023 Jan 23;13:1268. doi: 10.1038/s41598-023-28384-8 (PMC9871004; doi:10.1038/s41598-023-28384-8)
Supplement: Supplementary file 6 — Supplementary Information 6. [file 41598_2023_28384_MOESM6_ESM.pdf]

**Supplementary Table 6.** Y- STR allele frequencies in the population of North East Siberia (n = 9).								
**Allele**	**DYS19**	**DYS389I**	**DYS389II**	**DYS390**	**DYS391**	**DYS392**	**DYS393**	**DYS437**
**10**					0.667			
**11**					0.333			
**12**								
**13**	0.778	0.222					0.444	
**14**	0.222	0.667				0.556	0.556	0.222
**15**		0.111				0.444		0.778
**16**								
**17**								
**18**								
**19**								
**20**								
**21**								
**22**								
**23**				0.222				
**24**				0.556				
**25**				0.222				
**30**			0.556					
**31**			0.444					

DYS438	DYS439	DYS448	DYS456	DYS458	DYS635	GATA_H4	Genotype	DYS385a/b
0.333						0.222	12.19	0.222
0.667	0.556					0.333	13.17	0.111
						0.222	15.19	0.444
	0.333						16.16	0.111
	0.111			0.222		0.222	17.17	0.111
			0.556	0.222				
			0.333	0.111				
			0.111	0.444				
		0.222						
		0.222						
		0.222						
		0.333						
					0.889			
					0.111			
