# Supplementary material for: Bidirectional dispersals during the peopling of the North American Arctic
Source: Sci Rep. 2023 Jan 23;13:1268. doi: 10.1038/s41598-023-28384-8 (PMC9871004; doi:10.1038/s41598-023-28384-8)
Supplement: Supplementary file 7 — Supplementary Information 7. [file 41598_2023_28384_MOESM7_ESM.pdf]

Supplementary Table 7. Y- STR allele frequencies in the population of Tuva Republic (n = 24).

Allele	DYS19	DYS389I	DYS389II	DYS390	DYS391	DYS392	DYS393	DYS437	DYS438
**6**					0.042				
**10**					0.958				
**11**									0.958
**12**		0.042							0.042
**13**	1.000	0.917					0.958	0.958	
**14**		0.042				1.000	0.042	0.042	
**15**									
**16**									
**17**									
**18**									
**19**									
**20**									
**22**									
**23**				0.792					
**24**				0.208					
**30**			0.333						
**31**			0.667						

DYS439	DYS448	DYS456	DYS458	DYS635	GATA_H4	Genotype	DYS385a/b
						14.14	0.042
						15.15	0.042
0.083					0.958	15.16	0.625
0.792					0.042	15.17	0.083
0.125						16.16	0.167
						16,20	0.042
		0.958					
		0.042	0.208				
			0.125				
			0.625				
	0.875		0.042				
	0.125						
				0.917			
				0.083			
