# Supplementary material for: Bidirectional dispersals during the peopling of the North American Arctic
Source: Sci Rep. 2023 Jan 23;13:1268. doi: 10.1038/s41598-023-28384-8 (PMC9871004; doi:10.1038/s41598-023-28384-8)
Supplement: Supplementary file 9 — Supplementary Information 9. [file 41598_2023_28384_MOESM9_ESM.pdf]

Supplementary Table 9. Rst values for all pairwise populations comparisons (below diagonal) ar

	TUV	NSIB	BET	BAR	NALT	SALT	NWT
TUV	*	**<u>p<0.00001</u>**	**<u>p<0.00001</u>**	**<u>p<0.00001</u>**	**<u>p<0.00001</u>**	**0.00129**	**<u>p<0.00001</u>**
NSIB	**<u>1.0045</u>**	*	0.46867	0.06336	**0.00287**	**<u>p<0.00001</u>**	**0.00822**
BET	**<u>0.5870</u>**	0.0000	*	**0.00406**	**<u>0.00059</u>**	**<u>p<0.00001</u>**	**<u>p<0.00001</u>**
BAR	**<u>1.4492</u>**	0.1163	**0.1222**	*	**<u>p<0.00001</u>**	**<u>p<0.00001</u>**	0.16592
NALT	**<u>1.2713</u>**	**0.1971**	**<u>0.1322</u>**	**<u>0.4808</u>**	*	**<u>p<0.00001</u>**	**<u>p<0.00001</u>**
SALT	**0.1475**	**<u>0.9533</u>**	**<u>0.6368</u>**	**<u>1.3434</u>**	**<u>1.3953</u>**	**<u>*</u>**	**<u>p<0.00001</u>**
NWT	**<u>1.4426</u>**	**0.2467**	**<u>0.2331</u>**	0.0221	**<u>0.6941</u>**	**<u>1.2186</u>**	*
ESER	**<u>1.7305</u>**	**<u>0.3361</u>**	**<u>0.3205</u>**	**0.0714**	**<u>0.8302</u>**	**<u>1.5651</u>**	**0.0538**
WSER	**<u>1.3066</u>**	0.1325	**0.1820**	0.0731	**<u>0.6096</u>**	**<u>1.1152</u>**	**0.0992**
KUJ	**<u>1.1944</u>**	0.0449	**0.0955**	**0.1029**	**<u>0.3846</u>**	**<u>1.0455</u>**	**<u>0.1810</u>**
QAA	**<u>1.9386</u>**	**0.2726**	**<u>0.3170</u>**	0.0652	**<u>0.8302</u>**	**<u>1.6902</u>**	**0.0834**
QEQ	**<u>2.3465</u>**	0.1743	**0.2127**	0.0000	**<u>0.9284</u>**	**<u>1.8253</u>**	0.0000

Values in bold = Significant at the 0.05 significance level.

Values underlined in bold = Significant at the 0.00076 significance level (Bonferroni correction).

ESER	WSER	KUJ	QAA	QEQ	
**p<0.00001**	**p<0.00001**	**p<0.00001**	**p<0.00001**	**p<0.00001**	
**0.00030**	0.06247	0.19493	**0.00644**	0.05524	
**p<0.00001**	**0.00178**	**0.02831**	**p<0.00001**	**0.01257**	
**0.01505**	0.05653	**0.04287**	0.05099	0.44312	
**p<0.00001**	**p<0.00001**	**p<0.00001**	**p<0.00001**	**p<0.00001**	
**p<0.00001**	**p<0.00001**	**p<0.00001**	**p<0.00001**	**p<0.00001**	
**0.02584**	**0.02237**	**0.00713**	**0.02604**	0.47322	
*	0.21245	**0.01040**	0.21196	0.75537	
0.0150	*	0.37165	0.26997	0.53599	
**0.1443**	0.0000	*	0.05069	0.17840	
0.0128	0.0123	0.1042	*	0.96268	
0.0000	0.0000	0.0584	0.0000	*	
