# Supplementary figures and images for: Bidirectional dispersals during the peopling of the North American Arctic
Source: Sci Rep. 2023 Jan 23;13:1268. doi: 10.1038/s41598-023-28384-8 (PMC9871004; doi:10.1038/s41598-023-28384-8)

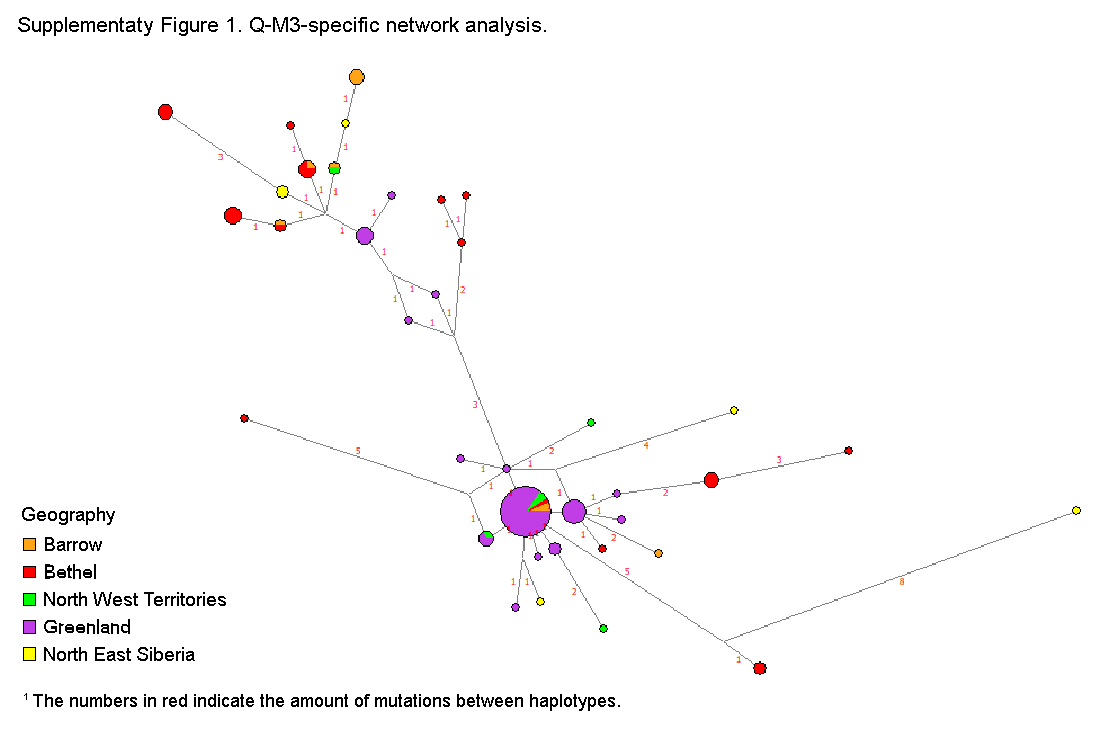

Supplement: Supplementary file 12 — Supplementary Information 12. [file 41598_2023_28384_MOESM12_ESM.tif]

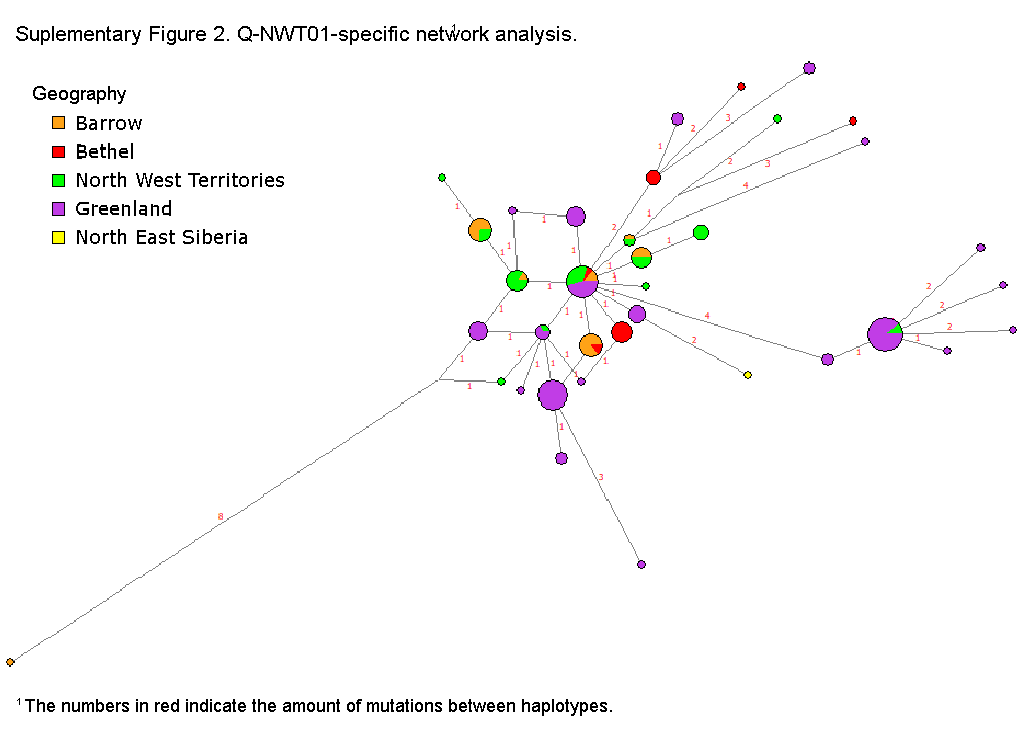

Supplement: Supplementary file 13 — Supplementary Information 13. [file 41598_2023_28384_MOESM13_ESM.tif]
